# Neurological examination course in an interactive webinar as a solution during a pandemic. An overview of the implementation, optimization as well as critical considerations

**DOI:** 10.3205/zma001405

**Published:** 2021-01-28

**Authors:** C. Oster, I. Farhood, S. Klebe, C. Kleinschnitz, Lorenz Peters

**Affiliations:** 1 Universitätsklinikum Essen, Klinik für Neurologie, Essen, Germany

**Keywords:** webinar, online bedside-teaching, neurology, COVID-19 pandemic

## Abstract

**Situation: **The COVID-19 pandemic made the traditional bedside teaching inaccessible for medical students.

**Problem: **Within a short period of time, established bedside teaching concepts had to be converted into online formats to meet the requirements of the health authorities.

**Approach: **The Department of Neurology at the University Hospital Essen transformed the examination course in the 5^th^ clinical semester into a live stream, taking into account data protection guidelines. This enabled students to participate from a distance, allowing them to take the medical history from a patient and to interact with the medical examiners. Thus, this concept goes beyond the video-based formats of the examination course.

**Optimization: **During the course, we performed online evaluations to ensure an immediate feedback from the students. This enabled us to implement ongoing changes that had a positive impact on the course format, for example using better equipment to ensure a better video and audio quality. In the future, we hope to create a clinic's own online channel to further increase data security.

## Zusammenfassung

**Situation: **Die COVID-19-Pandemie führte dazu, dass der praktische Teil der Lehre nicht in gewohnter Form stattfinden konnte.

**Problemstellung:** Innerhalb kürzester Zeit mussten etablierte Präsenzkonzepte auf Onlineformate umgestellt werden, um den gesundheitsbehördlichen Vorgaben gerecht zu werden.

**Lösungsansätze:** Die Klinik für Neurologie am Universitätsklinikum Essen führte unter Berücksichtigung der Datenschutzrichtlinien den praktischen Teil der Lehre im 5. klinischen Semester als live übertragenen Untersuchungskurs durch, was eine Partizipation der Studierenden bei der Anamnese und eine Interaktion mit den Dozierenden ermöglichte. Somit geht dieses Konzept über die videobasierten Formate des Untersuchungskurses hinaus. Hierbei wurden Patientinnen und Patienten nach deren Einverständnis mit Hilfe der online zugeschalteten Studierenden vor der Kamera anamnestiziert und untersucht.

**Optimierungsmöglichkeiten: **Im Verlauf wurde die technische Ausstattung deutlich verbessert, um bessere Bild- und Tonqualität zu gewährleisten. Für Lob und Kritik bestand ein enger Austausch mit den Studierenden. Ziel ist in Zukunft ein klinikeigener Onlinekanal um die Datensicherheit weiter zu erhöhen.

## Situation

After the outbreak of the COVID-19 pandemic, infection control regulations, which were based primarily on contact restrictions, posed great challenges for teaching. Hence, the neurological examination course, which is part of a bedside-teaching program, could not be held in the Neurology Department at the University Hospital Essen in the summer semester 2020 to protect students, staff and patients. 

## Problem description

The established presence concepts of teaching had to be converted into an online based alternative concept within a few weeks. Especially the conversion of the practical part proved to be challenging [1]. However, the proximity to the patient or clinical case is an essential part of teaching [2]. Thus, a concept with technical solutions had to be developed, to guarantee both, the required safety distance and the most possible amount of participation. 

## Solution approaches

The main teaching part of neurology takes place at the University Hospital Essen in the 5^th^ clinical semester. In addition to the free provision of an e-book on neurological examination with video collection, we decided to offer live streamed examination courses with subsequent case discussions and a repetition of the neuroanatomical basics. They were based on six thematically structured sections, from the neurological anamnesis to the examination of the different neurological systems. 

## Procedure

**Figure 1:**
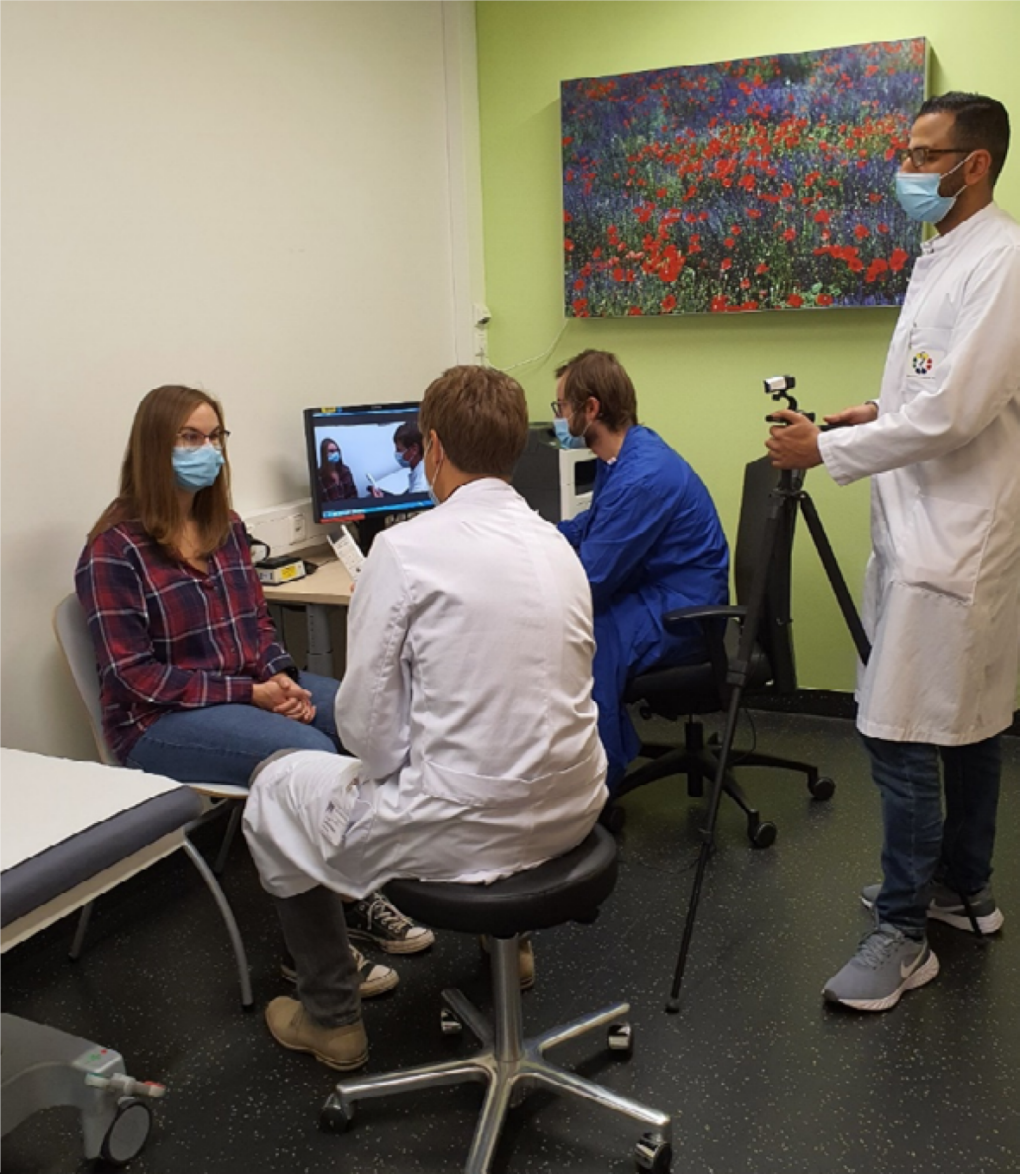
Representation of the examination situation on a patient during the anamnesis with video recording and taking over of the chat function at the computer.

The overall organization of the course was accompanied by two teaching assistants assigned to the semester who were responsible for the technical provision, the instruction of lecturers and the communication with the students. The assistants were also responsible for providing an in-patient with thematically matching symptoms for every course. It was important to select patients who had a sufficient understanding of the limitations of a webinar using commercial software solutions. The patients gave their written consent to be anamnestized and examined live in front of the camera. A certain anonymity was achieved by wearing a face mask continuously except for a few situations. The students were explicitly reminded to maintain confidentiality and that screencasting of the physical examination was prohibited. During the online course, students first took the patients history by switching on their microphone. Subsequently, a systematic examination was conducted by the assistant with another colleague operating the camera and an instructed student checking the participant lists on the computer, coordinating requests to speak and supervising the chat (see figure 1 (Fig. 1)). The webinar was supervised by a senior physician who joined the course online. Following the practical part, he or she led the case discussion and responded to questions or possible mistakes during the examination. In addition, the students were encouraged to follow up on important examination steps at home. As proof of their performance the students then prepared and submitted a short case summary.

## Technical solution

In the preparation of the course, an examination room was selected that offered fast Internet access and sufficient space for recording the gait pattern. Due to the lack of faculty-internal tested software solutions for video conferencing, we decided to use the software Zoom^®^ (Company: Zoom Video Communications; San José; USA) for which a license was purchased. The main reasons were the user-friendliness and stability of the transmission. Despite the much-discussed limitations [3], we aimed to ensure a sufficient level of data security. The students received their access code with changing passwords via their personalized learning platform account just immediately before the appointment. The students then had to give their full name with their attendance being monitored.

## Optimization

In addition, voluntary online evaluations were conducted after each block in which 63 of 155 students participated. The overall satisfaction with the course was rated as good with 3.9 out of 5 points, whereby the satisfaction with the learning progress was rated 3.8 out of 5 for the neurological anamnesis and 3.7 out of 5 for the examination technique. The structure of the course was rated positive with 4.1 out of 5. In the beginning mainly technical conditions such as sound or image quality were criticized, which were quickly optimized (see table 1 (Tab. 1)). There were limitations for a few students with regard to their technical requirements. Loan devices or protected access, e.g. in libraries, could help here. 

## Discussion

The live transmitted examination course is an attempt in times of the pandemic to enable the participation of students in addition to the existing video formats, whereby comparative studies would be desirable with regard to verify the learning progress [4]. Furthermore, the teaching concept of “See one, do one” could be raised to the four-step model according to Peyton, which could be transferred in parts to the concept described here [5]. In addition to the participation control by sending in the case description according to the SOAP scheme, it would also be possible for the students to hand in short self-filmed examination videos. Moreover, a video service hosted by the faculty would be desirable to counteract the ongoing discussion about data security with regard to commercial software use. 

## Authorship

Oster C. and Farhood I. share the first authorship. 

## Competing interests

The authors declare that they have no competing interests. 

## Figures and Tables

**Table 1:**
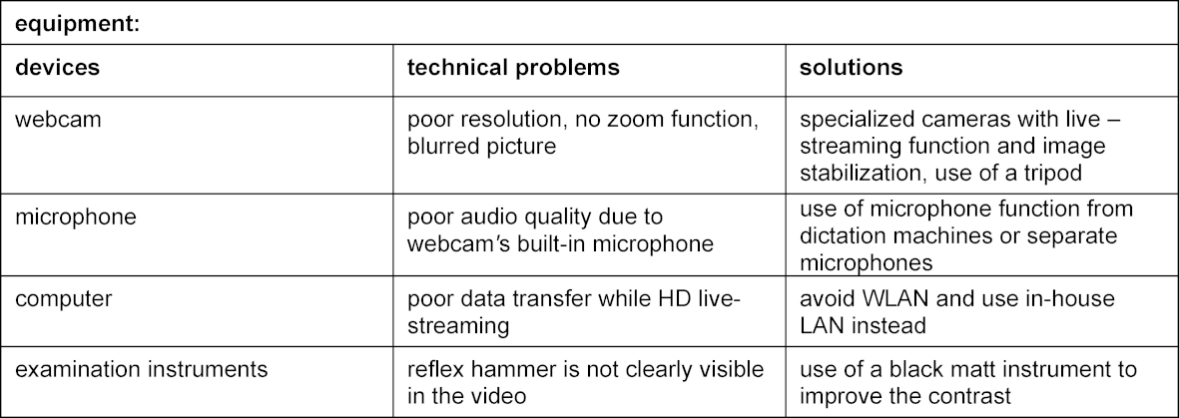
Overview of technical equipment and solutions for optimization
